# CONCEPTT: Continuous Glucose Monitoring in Women with Type 1 Diabetes in Pregnancy Trial: A multi-center, multi-national, randomized controlled trial - Study protocol

**DOI:** 10.1186/s12884-016-0961-5

**Published:** 2016-07-18

**Authors:** Denice S. Feig, Elizabeth Asztalos, Rosa Corcoy, Alberto De Leiva, Lois Donovan, Moshe Hod, Lois Jovanovic, Erin Keely, Craig Kollman, Ruth McManus, Kellie Murphy, Katrina Ruedy, J. Johanna Sanchez, George Tomlinson, Helen R. Murphy, on behalf of the CONCEPTT Collaborative Group

**Affiliations:** Mt Sinai Hospital, Toronto, Ontario Canada; Lunenfeld-Tanenbaum Research Institute, Toronto, Ontario Canada; Department of Medicine, University of Toronto, Toronto, Canada; Sunnybrook Health Sciences Centre, Toronto, Ontario Canada; Hospital de la Santa Creu i Sant Pau, Barcelona, Spain; CIBER-BBN, Zaragoza, Spain; University of Calgary, Calgary, Alberta Canada; Helen Schneider Hospital for Women, Rabin Medical Center, Petah Tikva, Israel; 30-1 Barranca Avenue, Santa Barbara, California USA; The Ottawa Hospital, Riverside Campus, Ottawa, Ontario Canada; Jaeb Center For Health Research, Tampa, Florida USA; St. Joseph Health Care London, London, Ontario Canada; Sunnybrook Research Institute, Toronto, Ontario Canada; University Health Network, Toronto General Hospital, Toronto, Ontario Canada; Institute of Metabolic Science, Cambridge University Hospitals NHS Foundation Trust, Cambridge, UK; Norwich Medical School, University of East Anglia, Norwich, UK; Department of Medicine, Leadership Sinai Centre for Diabetes, Mt Sinai Hospital, 60 Murray St. #5027, Toronto, Ontario M5T 3 L9 Canada

**Keywords:** Diabetes mellitus type 1, Pregnancy, Preconception, Continuous glucose monitoring, Randomized controlled trial

## Abstract

**Background:**

Women with type 1 diabetes strive for optimal glycemic control before and during pregnancy to avoid adverse obstetric and perinatal outcomes. For most women, optimal glycemic control is challenging to achieve and maintain. The aim of this study is to determine whether the use of real-time continuous glucose monitoring (RT-CGM) will improve glycemic control in women with type 1 diabetes who are pregnant or planning pregnancy.

**Methods/design:**

A multi-center, open label, randomized, controlled trial of women with type 1 diabetes who are either planning pregnancy with an HbA1c of 7.0 % to ≤10.0 % (53 to ≤ 86 mmol/mol) or are in early pregnancy (<13 weeks 6 days) with an HbA1c of 6.5 % to ≤10.0 % (48 to ≤ 86 mmol/mol). Participants will be randomized to either RT-CGM alongside conventional intermittent home glucose monitoring (HGM), or HGM alone. Eligible women will wear a CGM which does not display the glucose result for 6 days during the run-in phase. To be eligible for randomization, a minimum of 4 HGM measurements per day and a minimum of 96 hours total with 24 hours overnight (11 pm-7 am) of CGM glucose values are required. Those meeting these criteria are randomized to RT- CGM or HGM. A total of 324 women will be recruited (110 planning pregnancy, 214 pregnant). This takes into account 15 and 20 % attrition rates for the planning pregnancy and pregnant cohorts and will detect a clinically relevant 0.5 % difference between groups at 90 % power with 5 % significance. Randomization will stratify for type of insulin treatment (pump or multiple daily injections) and baseline HbA1c. Analyses will be performed according to intention to treat. The primary outcome is the change in glycemic control as measured by HbA1c from baseline to 24 weeks or conception in women planning pregnancy, and from baseline to 34 weeks gestation during pregnancy. Secondary outcomes include maternal hypoglycemia, CGM time in, above and below target (3.5–7.8 mmol/l), glucose variability measures, maternal and neonatal outcomes.

**Discussion:**

This will be the first international multicenter randomized controlled trial to evaluate the impact of RT- CGM before and during pregnancy in women with type 1 diabetes.

**Trial registration:**

ClinicalTrials.gov Identifier: NCT01788527 Registration Date: December 19, 2012.

**Electronic supplementary material:**

The online version of this article (doi:10.1186/s12884-016-0961-5) contains supplementary material, which is available to authorized users.

## Background

Despite all efforts, women with type 1 diabetes in pregnancy continue to have increased rates of adverse pregnancy outcomes. Women aiming for optimal glycemic control are at substantially increased risk of severe hypoglycemia (episode of low blood glucose requiring third party assistance) as well as pregnancy related complications of gestational hypertension, preeclampsia and delivery by caesarean section. Infants of mothers with diabetes face increased risk of preterm delivery, macrosomia, neonatal hypoglycemia, hyperbilirubinemia, respiratory distress and neonatal intensive care unit admissions. Macrosomia itself is associated with shoulder dystocia, birth injury, asphyxia and death. In a study of over 1,000,000 deliveries in Ontario, Canada, the rates of perinatal mortality and congenital anomalies among women with pre-existing diabetes in pregnancy were found to be approximately twice the rates of women without diabetes [1].

Numerous studies have shown that adverse pregnancy outcomes can be reduced with improved glycemic control. Pre-pregnancy care has been shown to assist women to improve glycemic control during the crucial period of organogenesis, and has been associated with reduced rates of adverse pregnancy outcomes including major congenital malformation, stillbirth and neonatal death. However, even motivated women who attend pre-pregnancy clinics still struggle to achieve and maintain optimal glycemic control [2].

CGM systems contain a subcutaneous glucose-sensing device which measures interstitial glucose and provide detailed information about the frequency and duration of glucose excursions, which is either unavailable to the user at the time of collection but available after (masked CGM) or available at the time (RT-CGM). One study comparing conventional home glucose monitoring (HGM) with masked CGM, found that CGM detected substantial hyperglycemia (>3 hours/day) and overnight hypoglycemia (1–4 hours) missed by conventional glucose monitoring [3]. Another study demonstrated that pregnant women with type 1 diabetes are still far from achieving the recommended glucose control target range of 3.9–7.8 mmol/l [4]. During the first trimester, masked CGM demonstrated that women spent 10–12 h per day hyperglycemic (>7.8 mmol/L) and 2–3 h hypoglycemic (<3.9 mmol/l). By the third trimester maternal hyperglycemia improved only slightly even with frequent antenatal clinic visits.

RT- CGM use provides additional information for the user to consider when adjusting diet, activity and insulin doses. A systematic review in non-pregnant adults, demonstrated that RT- CGM use is associated with modest improvements in glycemic control (a mean HbA1c reduction of 0.3 %), with maximal impact (up to 1.0 % reduction in HbA1c) in those with poor glycaemic control who use CGM at least 6 days per week [5]. However data from two randomized trials in pregnancy are conflicting. In a UK trial of 71 women with type 1 and type 2 diabetes, randomized to wearing a masked CGM every 4–6 weeks compared to standard care with HGM, the use of the CGM was associated with both reduced HbA1c (0.6 %) and reduced risk of macrosomia (OR 0.36, 95 % CI 0.13-0.98) [6]. A subsequent Danish trial of 154 women, randomized to use RT-CGM intermittently (six days x five times) or standard care with HGM found no difference in glycemic control or neonatal outcomes [7]. This may have been because women had good glycaemic control at baseline and were not particularly compliant with RT- CGM, with only 60 % of women using it intermittently. A systematic review thus concluded that more research is needed to identify the most effective techniques of blood glucose monitoring in pregnant women [8].

The aim of this study is to determine whether the use of continuous RT- CGM will improve glycemic control in women with type 1 diabetes who are a) planning pregnancy and b) in early in pregnancy, without substantially increasing the rate of hypoglycemia.

## Methods/design

### Overall study design

**Fig. 1:**
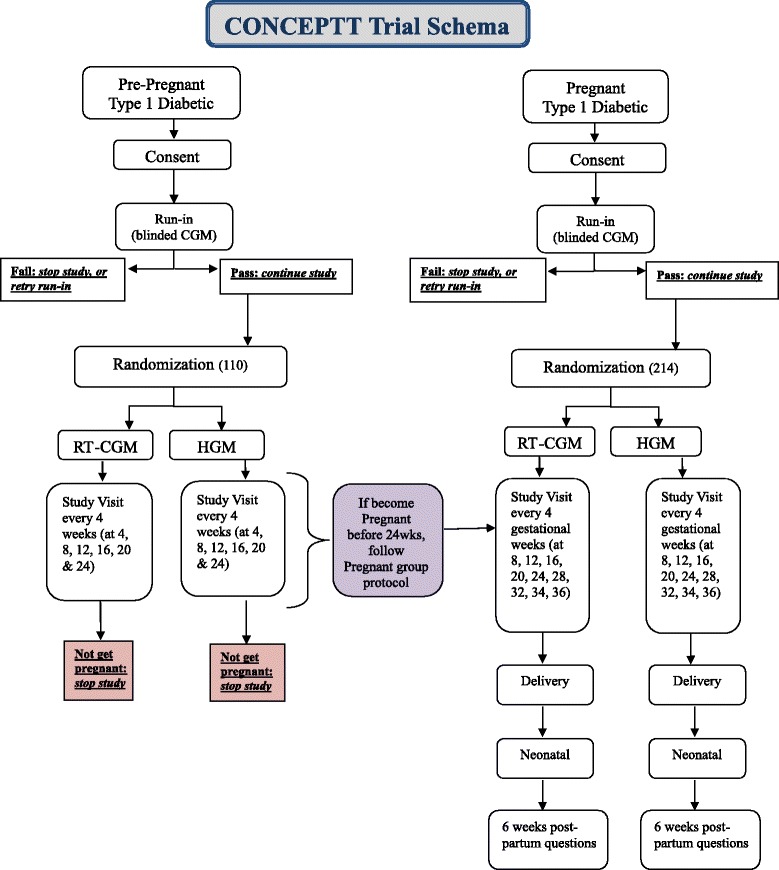
CONCEPTT Flow Diagram

CONCEPTT is a multicenter, randomized, open label, controlled trial with an intention-to-treat analysis of two parallel trials: one trial in women planning pregnancy, and one in women in early pregnancy. Thirty trial centers are located across six countries: Canada (11), UK (15), Spain (1), Italy (1), USA (1) and Ireland (1). Women with type 1 diabetes in pregnancy who are ≤13 weeks 6 days gestation with an HbA1c of 6.5 % to ≤10.0 % (48 to ≤86 mmol/mol), and women with type 1 diabetes planning pregnancy with an HbA1c of 7.0 % to ≤10.0 % (53 to ≤86 mmol/mol), will be eligible for the run-in phase (see Fig. 1). The run-in incorporates a 6-day period during which women wear a masked CGM (Medtronic iPro®2 Professional CGM with Enlite2 sensor) to ensure that they can tolerate wearing a CGM device. Women who pass the run-in (>96 hours total with ≥24 hours overnight [11 pm-7 am] of CGM data and at least 4 HGM measurements per day) are eligible for randomization. Eligible women are randomized to CGM (Medtronic MiniMed Guardian®, Medtronic MiniMed Paradigm® Veo™ or Medtronic MiniMed® 640G system as per participant insulin delivery method) along with usual HGM, or continue HGM without CGM. The primary outcome is the change in HbA1c from baseline to 24 weeks or conception in women planning pregnancy, and from baseline to 34 weeks gestation in women who are pregnant.

### Primary research questions

1. Among women with type 1 diabetes who are planning pregnancy, does the addition of RT-CGM to HGM improve glycemic control, as measured by change in HbA1c from randomization to 24 weeks (or at conception), when compared to a standard regimen of HGM without RT-CGM?
2. Among pregnant women with type 1 diabetes, does the addition of RT-CGM to a standard regimen of HGM improve glycemic control, as measured by change in HbA1c from randomization in early pregnancy to 34 weeks gestation (or if pregnancy loss, latest measurement), when compared to a standard regimen of HGM without RT-CGM?

### Eligibility criteria

To be eligible for the trial, participants must meet the following criteria:

- Clinical diagnosis of type 1 diabetes and using daily insulin therapy for at least one year
- Age 18–40 years
- Intensive insulin regimen involving either an insulin pump or multiple daily injections (MDI) of insulin
- No expectation that the participant will be moving out of the area during the next year

In addition, specific eligibility criteria apply for the pre-pregnant and pregnant cohorts:

- *Pre-pregnant cohort:*
  - Participants are planning pregnancy and wish to optimise glycemic control before conception

- *Pregnant cohort:*
  - Pregnancy gestation ≤13 weeks, 6 days at time of randomization
  - Live, singleton fetus
  - Ultrasound (US) done to confirm gestational age, viability and to rule out a multiple pregnancy.

Gestational age will be based on the last menstrual period (LMP) provided (±5 days discrepancy with US dates in the first trimester. If more than 5 days discrepancy from US date, or LMP not known, then US dates used).

### Exclusion criteria

- Type 2 diabetes
- Gestational diabetes
- Regular home user of real-time CGM in the previous 3 months
- Previous participation in the trial
- Estimated GFR <60 ml/min/1.73
- The presence of a significant medical disorder or use of a medication such as oral glucocorticoids that in the judgment of the investigator will affect wearing of the sensors or completion of the protocol.
- Inpatient psychiatric treatment in the past 6 months
- Participants using premixed fixed doses of insulin

In addition, specific exclusion criteria apply to the pre-pregnant and pregnant cohorts:

- *Pre-pregnancy cohort:*
  - HbA1c <7.0 % (<53 mmol/mol) or >10.0 % (>86 mmol/mol)

- *Pregnancy cohort:*
  - First HbA1c in pregnancy < 6.5 % (48 mmol/mol) or current HbA1c > 10.0 % (86 mmol/mol)
  - Known higher order pregnancies (twins, triplets, etc.).
  - Known fetal anomaly.

### Recruitment

Clinicians involved in the care of women with type 1 diabetes (pre-pregnant and pregnant) determine whether a patient meets the eligibility criteria and ask if she is interested in the trial. Interested patients are given a participant information sheet and time to consider taking part. Women who participate are asked to provide written informed consent prior to commencing the run-in period. Baseline data including diabetes history, current diabetes management and obstetric history are obtained. A standard physical exam (vital signs, and height and weight measurements) is performed and the participants complete validated questionnaires including the Blood Glucose Monitoring System Rating Questionnaire, the Problem Areas in Diabetes Questionnaire, the SF-12 and the Hypoglycemia Fear Survey II. UK participants are invited to complete a three day food diary.

### Run-in

The participant wears a masked CGM for 6 days. This provides a baseline assessment of glycemic control and allows participants to wear the CGM before making a decision about whether or not to continue to be randomized. The participant must test using the HGM at least 4 times per day and wear the CGM for at least 5 days, obtaining at least 96 h of glucose values and have at least 24 h of values between 23.00–07.00 h to qualify for randomization. The run-in CGM data are downloaded for evaluation by an independent data coordinating center (Jaeb Center For Health Research Inc, Tampa, Florida, USA) and not reviewed by participants or their clinicians. Should the first run-in be unsuccessful (due to insufficient CGM data), a repeat CGM can be obtained.

### Randomization

Participants who pass the run-in phase are randomized to either RT-CGM in addition to their standard HGM or HGM without CGM. The randomization process is run separately for the pre-pregnant and pregnant cohorts.

The participant’s random group assignment is determined via the Centre for Mother, Infant, and Child Research trial website. They construct a master randomization list using a permuted block design. The participants are stratified by mode of insulin therapy (pump vs. MDI) and baseline HbA1c (for pregnant group: <7.5 % or 58 mmol/mol vs. ≥7.5 % or 58 mmol/mol; for pre-pregnant group: <8.0 % or 64 mmol/mol vs. ≥8.0 % or 64 mmol/mol). Within strata, allocation are assigned in a ratio of 1:1 with random block sizes.

### Intervention

#### RT-CGM group

Participants randomized to the RT-CGM group are instructed to use the device on a daily basis. Participants are taught to insert the sensor device, perform calibration tests, use insulin dose adjustment algorithms (see Supplementary Pages for example) and to make changes to their insulin regimen based on the data from the RT-CGM and HGM. Additional HGM glucose measurements may be performed anytime, particularly prior to making management decisions based on RT-CGM values.

#### HGM group

Participants randomized to the HGM group are asked to perform at least 7 finger stick blood glucose measurements per day. Participants are taught to use insulin dose adjustment algorithms and to make changes to their insulin regimen based on data from the HGM.

#### Follow-up visits and data collection

Visits take place every 4 weeks after randomization. For the prepregnant cohort, the final visit will be at 24 weeks following randomization or at the time pregnancy is confirmed. For the pregnancy cohort, the final visit will be at 34 weeks gestation. At each visit weight, blood pressure, insulin type and dose are collected. Glucose and insulin data are reviewed along with episodes of severe hypoglycemia. Recommendations regarding diabetes management are made by treating clinicians in routine antenatal clinics. For the RT-CGM group, the RT-CGM data are downloaded and the participant’s skin is assessed. For the HGM groups, a masked CGM is used twice: for 6 days at 12 and 24 weeks in the pre-pregnant cohort and for 6 days at 24 and 34 weeks gestation in the pregnant cohort. Data from the masked CGM is downloaded to the Jaeb Center for independent review. The RT-CGM group have their RT-CGM data downloaded to the Jaeb Center for independent review 7–10 days after 12 and 24 weeks in the pre-pregnant cohort, and 24 and 34 weeks gestation in the pregnant cohort. Final questionnaires and food diaries for UK participants are repeated at 24 weeks in the pre-pregnant cohort or at time of conception, and at 34 weeks gestation in the pregnant cohort.

#### Bloods

Bloods are drawn for HbA1c at randomization, and at 12 and 24 weeks in the pre-pregnant cohort, and at 24 and 34 weeks gestation in the pregnant cohort. Biorepository bloods are drawn in those that consent, at randomization and at 34 weeks gestation for the pregnant cohort only.

#### Delivery

At delivery, cord blood is taken for blood gases (done locally) and C-peptide. If the participant consents, cord blood biorepository sample is also collected for future research. Data is collected regarding delivery and neonatal outcomes. Fetal anthropometrics are done within the first 72 h after delivery. At 6 weeks postpartum data is collected regarding neonatal wellbeing and maternal satisfaction with participating in the trial.

Women that are in the pre-pregnant cohort who become pregnant, continue in the treatment arm they were randomized to, and are followed in a similar fashion to the pregnant cohort. However, their data are analyzed separately from the pregnant cohort.

### Primary outcome

#### Pre-pregnant cohort

The primary outcome is glycemic control as measured by a change in HbA1c from randomization to 24 weeks. If the participant becomes pregnant before 24 weeks, her final HbA1c is measured post-confirmation of a positive pregnancy test.

#### Pregnant cohort

The primary outcome is glycemic control as measured by a change in HbA1c from randomization to 34 weeks gestation. In women who do not progress to 34 weeks gestation, the latest measured HbA1c is used to contribute to the primary outcome.

### Secondary outcomes

#### Pre-pregnant cohort

- CGM time in target at baseline, 12 and 24 weeks.
- HbA1c at baseline, 12 and 24 weeks.

#### Pregnant cohort

- CGM time in target at baseline, 24 and 34 weeks gestation.
- HbA1c at baseline, 24 and 34 weeks gestation
- Incidence of gestational hypertension/preeclampsia
- Caesarean section: pre-labour and intrapartum
- Gestational weight gain (randomization to 36 weeks)

#### Pre-pregnant and pregnant cohorts

- Area under the curve (AUC)
  - AUC for CGM glucose > 7.8 mmol/l
  - AUC for CGM glucose > 6.7 mmol/l
  - AUC for CGM glucose < 3.5 mmol/L
  - AUC for CGM glucose < 2.8 mmol/L
- Hypoglycemia
  - Episodes of ‘severe hypoglycemia’ requiring third party assistance
  - Mild-moderate episodes of hypoglycemia from CGM data <3.5 mmol/L (mild) and <2.8 mmol/L (moderate) for 20 min duration
  - Nocturnal hypoglycemia: CGM glucose <3.5 mmol/L (mild) and <2.8 mmol/L (moderate) for 20 min duration between 23.00–07:00 h
- Measures of glucose variability:
  - Mean amplitude of glycemic excursions
  - SD of CGM measurements
  - Mean absolute rate of change of CGM based on one week of sensor values
- Length of hospital stay associated with delivery
- Questionnaires
- Insulin requirements
- Safety outcome:
  - A substantial increase in hypoglycemia will be defined as >10 % increase in hypoglycemic episodes (<3.5 mmol/L for at least 20 min duration) over and above the HGM group.

Infant Outcomes.

- Birth weight:
  - Infant birth weight >90th centile using national growth curves
  - Infant birth weight >90th centile using customized centiles
  - Infant birth weight <10th centile, using national growth curves
  - Infant birth weight <10th centile using customized centiles
  - Infant birth weight ≥4 kg
- Pregnancy loss: miscarriage, stillbirth, neonatal death (death ≤28 days of life)
- Preterm birth (<37 weeks and early preterm <34 weeks)
- Birth injury
- Shoulder dystocia
- Neonatal hypoglycemia
- Hyperbilirubinemia
- Respiratory distress
- High level neonatal care > 24 h
- Cord blood gas pH < 7.0
- Hyperinsulinemia (using cord c-peptide)
- Composite fetal outcome: Pregnancy loss: miscarriage, stillbirth, neonatal death (death ≤28 days of life), birth injury, neonatal hypoglycemia, hyperbilirubinemia, respiratory distress, high level neonatal care > 24 h.
- Sum of skin-folds >90th percentile for gestational age – triceps, sub scapular, biceps and suprailiac skin-folds
- Anthropometric measures - infant birth weight, head circumference, chest circumference, abdominal circumference, left and right upper-arm circumference, crown-heel length, crown-rump length
- Length of hospital stay until first discharge home

#### Statistical analysis

Primary Outcome: The primary analysis will compare the treatment groups on the 24-week HbA1c for the pre-pregnant cohort and on the 34-week HbA1c for the pregnant cohort, controlling for baseline HbA1c in an analysis of covariance that includes the treatment modality (pump/MDI) and strata used in randomization as covariates. We will obtain point and interval estimates of the treatment effect (the mean adjusted difference in follow-up HbA1c between treated and control groups) and also test the null hypothesis that the treatment effect is zero. The primary analysis will follow the intention-to-treat principle with all participants analyzed in the group to which they were randomized, regardless of actual sensor wear. In the analysis of the pre-pregnant cohort, in women who become pregnant before 24 weeks, the final outcome will be a measurement of HbA1c taken post-confirmation of pregnancy. In the analysis of the pregnant cohort, in women who do not reach 34 weeks gestation, the last HbA1c taken prior to 34-weeks will be used for the primary outcome. If important covariates remain imbalanced between treatment groups despite the stratified randomization, these covariates will be added to the regression model and the difference between adjusted and unadjusted estimates will be examined to assess the impact of this imbalance. Multiple imputation using earlier HbA1c measurements will be used to deal with HbA1c values that are missing at the final assessment.

#### Sample size estimation

The trial will include 324 participants, with 110 in the pre-pregnant cohort (women planning pregnancy), and 214 in the pregnant cohort.

In both cohorts, the sample size is based on a clinically relevant difference in HbA1c of 0.5 %. For pregnant women, a cross-sectional standard deviation (SD) of 1.1 was used, as it is towards the upper limit of published HbA1c SD values ([9–11]. In pre-pregnant women, a SD value of 0.8 was used, based on reported SD values in a trial of CGM in young adults [12]. As this latter study also reported the SD of change over 26 weeks, it was possible to compute the correlation between repeated measurements. In the various study groups, these correlations ranged from 0.4 to 0.7. To be conservative, we used the lower value of 0.4 in our sample size calculations. In both cohorts (pre-pregnant and pregnant), sample size was computed for an analysis of covariance with the final HbA1c as the outcome and baseline HbA1c and treatment group as predictors. Power was set at 90 % and the two-tailed significance level was set at 5 %.

#### Data management

The continuous glucose monitoring data management and analyses will be handled by the Jaeb Center for Health Research, Tampa, Florida. All other data and statistical aspects will be handled by the Clinical Trials Services/Centre for Mother, Infant and Child Research, Toronto, Ontario, and the trial statistician.

#### Trial steering committee

A trial steering committee will be responsible for the conduct of the trial. They will meet by teleconference on a quarterly basis to review the progress of the trial or on an ad hoc basis should the need arise.

#### Safety considerations

A Data Safety Monitoring Board (DSMB) will be established and will include experts in or representatives of the fields of endocrinology, obstetrics, epidemiology, and clinical trials methodology. They will meet after the initial safety analysis is completed, which will be done after 50 % of the pregnant group has been recruited. Serious unanticipated adverse events will be reported to the DSMB should the need arise.

## Discussion

### Implications of the findings

We aim to evaluate the impact of RT- CGM on glycemic control in two groups of women, those who are planning pregnancy and women in early pregnancy. If we find an improvement, the use of the RT-CGM will be encouraged and potentially reimbursed. We may also be able to determine if RT-CGM is more helpful in certain subsets of participants (e.g. those using pump or MDI), and whether the use of RT-CGM will affect maternal and neonatal outcomes. If we do not find that RT-CGM is beneficial, then other technologies such as closed-loop insulin delivery, may be indicated to facilitate optimal glycemic control in type 1 diabetes pregnancies.

### Dissemination

A report will be written for the funding bodies and for peer-reviewed publication and will be disseminated to international lay and scientific audiences.

## Conclusion

Results from studies in non-pregnant populations suggest that CGM improves glycemic control. Results from two randomized studies performed during pregnancy are conflicting, one with and one without improved glycemic control. This is the first study to look at continuous use of RT- CGM both in women planning pregnancy and in women during early pregnancy. It will inform patients, caregivers, and funding agencies regarding the use of CGM in the pregnant woman with type 1 diabetes.

## Abbreviations

AUC, Area under the curve; DSMB, Data Safety Monitoring Board; HbA1c, Glycated haemoglobin; HGM, Home glucose monitoring; MDI, Multiple daily injections; RT-CGM, Real-time Continuous glucose monitoring; SD, Standard deviation

## Additional file

Additional file 1: Ethics Review Boards That Approved CONCEPTT. (DOCX 18 kb)
