# Erratum to: CONCEPTT: Continuous Glucose Monitoring in Women with Type 1 Diabetes in Pregnancy Trial: A multi-center, multi-national, randomized controlled trial - Study protocol

**DOI:** 10.1186/s12884-016-1036-3

**Published:** 2016-08-26

**Authors:** Denice S. Feig, Elizabeth Asztalos, Rosa Corcoy, Alberto De Leiva, Lois Donovan, Moshe Hod, Lois Jovanovic, Erin Keely, Craig Kollman, Ruth McManus, Kellie Murphy, Katrina Ruedy, J. Johanna Sanchez, George Tomlinson, Helen R. Murphy, on behalf of the CONCEPTT Collaborative Group

**Affiliations:** 1 Mt. Sinai Hospital, Toronto, Ontario Canada; 2 Lunenfeld-Tanenbaum Research Institute, Toronto, Ontario Canada; 3 Department of Medicine, University of Toronto, Toronto, Canada; 4 Sunnybrook Health Sciences Centre, Toronto, Ontario Canada; 5 Hospital de la Santa Creu i Sant Pau, Barcelona, Spain; 6 CIBER-BBN, Zaragoza, Spain; 7 University of Calgary, Calgary, Alberta Canada; 8 Helen Schneider Hospital for Women, Rabin Medical Center, Petah Tikva, Israel; 9 30-1 Barranca Avenue, Santa Barbara, CA USA; 10 The Ottawa Hospital, Riverside Campus, Ottawa, Ontario Canada; 11 Jaeb Center For Health Research, Tampa, FL USA; 12 St. Joseph Health Care London, London, Ontario Canada; 13 Sunnybrook Research Institute, Toronto, Ontario Canada; 14 University Health Network, Toronto General Hospital, Toronto, Ontario Canada; 15 Institute of Metabolic Science, Cambridge University Hospitals NHS Foundation Trust, Cambridge, UK; 16 Norwich Medical School, University of East Anglia, Norwich, UK; 17 Department of Medicine, Leadership Sinai Centre for Diabetes, Mt. Sinai Hospital, 60 Murray St. #5027, Toronto, Ontario M5T 3L9 Canada

## Erratum

After publication of the original article [1], it came to the authors’ attention that an incorrect affiliation was inadvertently added in the Acknowledgements section for the CONCEPTT Collaborative Group. The authors would like to amend the following statement in the CONCEPTT Collaborative Group section as follows: The correct affiliation for Julia Lowe and Anna Rogowsky should read Sunnybrook Health Sciences Centre, Toronto.
