# Supplementary material for: CONCEPTT: Continuous Glucose Monitoring in Women with Type 1 Diabetes in Pregnancy Trial: A multi-center, multi-national, randomized controlled trial - Study protocol
Source: BMC Pregnancy Childbirth. 2016 Jul 18;16:167. doi: 10.1186/s12884-016-0961-5 (PMC4950103; doi:10.1186/s12884-016-0961-5)
Supplement: Additional file 1: — Ethics Review Boards That Approved CONCEPTT. (DOCX 18 kb) [file 12884_2016_961_MOESM1_ESM.docx]

**Additional file 1**

Ethics Review Boards That Approved CONCEPTT

| **Site** | **Ethics Review Board** |
| --- | --- |
| Royal University Hospital, Saskatoon | University of Saskatoon Biomedical Research Ethics Board (Bio-REB) |
| Mt Sinai Hospital, Toronto | Mount Sinai Hospital Research Ethics Board |
| IWK Health, Halifax | IWK Research Ethics Board (IWK-REB) |
| Sunnybrook Health Sciences Centre, Toronto | Sunnybrook Health Sciences Centre Research Ethics Office |
| Kingston General Hospital | QUEEN'S UNIVERSITY HEALTH SCIENCES AND AFFILIATED TEACHING HOSPITALS RESEARCH ETHICS BOARD |
| The Ottawa Hospital | Ottawa Health Science Network Research Ethics Board |
| McMaster University Hospital, Hamilton | Hamilton Integrated Research Ethics Board |
| St Michael’s Hospital, Toronto | St Michael’s Research Ethics Office |
| St Joseph’s Hospital, London, Ontario | University of Western Ontario Health Sciences Research Ethics Board (HSREB) |
| Alberta Health Services – Calgary zone | University of Calgary Conjoint Health Research Ethics Board (CHREB) |
| CHU de Québec, Quebec City | Comité d’éthique de la recherche du CHU de Québec |
| CHUM, Montreal | Comité d’éthique de la recherche du CHUM |
| William Sansum Diabetes Center, Santa Barbara, California | Santa Barbara Hospital Cottage Hospital, Cottage Health System IRB |
| UK Ethics | Cambridge University Hospitals NHS Foundation Trust Research and Development Department |
| Niguarda ca' Granda Hospital, Milan | Azienda Ospedaliera Ospedale Niguarda Ca’ Granda COMITATO ETICO-SCIENTIFICO |
| Hospital De La Santa Creu I Sant Pau, Barcelona | Fundació de Gestió Sanitària de l’Hospital De La Santa Creu I Sant Pau Comitè Ètic D’investigació ClÍnica |
| Galway University Hospital | Merlin Park University Hospital Ospidéal na h-Ollscolie, Páirc Mheirlinne Galway University Hospitals Clinical Research Ethics Committee |
